# Data on daily fluoride intake based on drinking water consumption prepared by household desalinators working by reverse osmosis process

**DOI:** 10.1016/j.dib.2016.06.048

**Published:** 2016-07-02

**Authors:** Vahid Noroozi Karbasdehi, Sina Dobaradaran, Abdolhamid Esmaili, Roghayeh Mirahmadi, Fatemeh Faraji Ghasemi, Mozhgan Keshtkar

**Affiliations:** a Department of Environmental Health Engineering, Faculty of Health, Bushehr University of Medical Sciences, Bushehr, Iran; b The Persian Gulf Marine Biotechnology Research Center, The Persian Gulf Biomedical Sciences Research Institute, Bushehr University of Medical Sciences, Bushehr, Iran; c Systems Environmental Health, Oil, Gas and Energy Research Center, The Persian Gulf Biomedical Sciences Research Institute, Bushehr University of Medical Sciences, Bushehr, Iran

**Keywords:** Bushehr, Daily fluoride intake, Household desalinators, Reverse osmosis

## Abstract

In this data article, we evaluated the daily fluoride contents in 20 household desalinators working by reverse osmosis (RO)^1^ process in Bushehr, Iran. The concentration levels of fluoride in inlet and outlet waters were determined by the standard SPADNS method using a spectrophotometer (M501 Single Beam Scanning UV/VIS, UK). The fluoride content in outlet waters were compared with EPA and WHO guidelines for drinking water.

**Specifications Table**

**Table:** 

*Subject area*	*Chemistry*
*More specific subject area*	*Daily fluoride intake*
*Type of data*	*Table*
*How data was acquired*	*Spectrophotometer (M501 Single Beam Scanning UV/VIS, UK)*
*Data format*	*Raw, analyzed*
*Experimental factors*	*All water samples in plastic bottles were stored in a dark place at room temperature in their plastic containers until the fluoride analysis.*
*Experimental features*	*Evaluate the fluoride content in inlet and outlet water of household desalinators working by RO process*
*Data source location*	*Bushehr, Iran*
*Data accessibility*	*Data is with this article.*

**Value of the data**

• Data can be used as a base-line data for the fluoride content in drinking water prepared by household desalinators.
• Data shown here will be informative in computing fluoride daily intake by drinking water as well as food consumption.
• Data shown here can be useful for health policy makers by assigning prevention measures against adverse health effects of fluoride with considering fluoride intake by different sources.

## 1 Data

In the data, as shown in Table 1, the mean concentration levels of fluoride in inlet and outlet waters were 0.47 and 0.18 with a range of 0.32–0.63 and 0–0.47 mg/l respectively. The mean removal percent of fluoride by household desalinators was 63.7 with a range of 2.1–100%. As seen in Table 1, it shows that the mean value daily intakes of fluoride based on 2 liters daily drinking water consumption [1] reached 0.36 mg/day with a range of 0–0.96 mg/day.

## 2 Experimental design, materials and methods

This cross-sectional descriptive study was carried out using random sampling (In different areas of Bushehr). Samples were taken from inlet and outlet waters of household desalinators working by RO process between February and March 2016. A total number of 40 samples (Inlet and outlet waters) were taken from 20 household desalinators and analyzed for fluoride contents. For sampling, we used plastic containers. All bottles were stored in a dark place at room temperature until the fluoride analysis was made by the standard SPADNS method [2], [3], [4], [5], [6], [7], [8], [9], [10], [11], [12], [13], [14], [15] using a Spectrophotometer (M501 Single Beam Scanning UV/VIS, UK. The fluoride amounts of inlet and outlet waters were compared with EPA and WHO guidelines for drinking water. Finally daily fluoride intakes were calculated based on 2 liters daily drinking water consumption and concentration levels of fluoride in outlet waters.

## Figures and Tables

**Table 1:** Mean concentration levels of fluoride (mg/l) in inlet and outlet waters, removal percentage of fluoride by household desalinators, comparison with EPA and WHO guidelines for drinking water, and daily fluoride intakes.

**Different areas**	**Inlet**	**Outlet**	**Removal rate (%)**	**Daily intake (mg/day)**
*Bagh Zahra*	0.61	0.41	32.8	0.82
*Helali*	0.45	0	100	0
*Shekari*	0.63	0.31	50.8	0.62
*Khajeha*	0.49	0	100	0
*Bahonar*	0.51	0.07	86.3	0.14
*Solh-Abad*	0.50	0.06	88	0.12
*Sartol*	0.60	0.08	86.7	0.16
*Jabri*	0.47	0.42	10.7	0.84
*Rishehr*	0.51	0.16	68.6	0.32
*Sangi*	0.45	0	100	0
*Jofreh*	0.39	0.44	11.37	0.88
*Modares*	0.38	0	100	0
*City center*	0.47	0.48	2.1	0.96
*Davas*	0.53	0.41	22.7	0.82
*Ashouri*	0.45	0	100	0
*Emam Reza*	0.57	0.31	45.6	0.62
*Sabz Abad*	0.37	0.34	8.1	0.68
*Tangak*	0.33	0.03	90.1	0.06
*Bandargah*	0.32	0.03	90.6	0.06
*Bahmani*	0.33	0.07	78.8	0.14
*Minimum value*	0.32	0	2.1	0
*Maximum value*	0.63	0.48	100	0.96
*Mean value*	0.47	0.18	63.7	0.36
*Std. deviation*	0.092	0.178	36.6	0.37
*EPA standard*	–	2	–	–
*WHO standard*	–	1.5	–	–

Based on 2 liters daily drinking water consumption and concentration levels of fluoride in outlet waters.
